# Volatiles Mediated Interactions Between *Aspergillus oryzae* Strains Modulate Morphological Transition and Exometabolomes

**DOI:** 10.3389/fmicb.2018.00628

**Published:** 2018-04-04

**Authors:** Digar Singh, Choong H. Lee

**Affiliations:** Department of Bioscience and Biotechnology, Konkuk University, Seoul, South Korea

**Keywords:** *Aspergillus oryzae*, VOCs mediated interactions, sclerotia development, biochemical phenotypes, exometabolomes, twin-plate assembly

## Abstract

Notwithstanding its mitosporic nature, an improbable morpho-transformation state i. e., sclerotial development (SD), is vaguely known in *Aspergillus oryzae*. Nevertheless an intriguing phenomenon governing mold's development and stress response, the effects of exogenous factors engendering SD, especially the volatile organic compounds (VOCs) mediated interactions (VMI) pervasive in microbial niches have largely remained unexplored. Herein, we examined the effects of intra-species VMI on SD in *A. oryzae* RIB 40, followed by comprehensive analyses of associated growth rates, pH alterations, biochemical phenotypes, and exometabolomes. We cultivated *A. oryzae* RIB 40 (S1_VMI_: KACC 44967) opposite a non-SD partner strain, *A. oryzae* (S2: KCCM 60345), conditioning VMI in a specially designed “twin plate assembly.” Notably, SD in S1_VMI_ was delayed relative to its non-conditioned control (S1) cultivated without partner strain (S2) in twin plate. Selectively evaluating *A. oryzae* RIB 40 (S1_VMI_ vs. S1) for altered phenotypes concomitant to SD, we observed a marked disparity for corresponding growth rates (S1_VMI_ < S1)_7days_, media pH (S1_VMI_ > S1)_7days_, and biochemical characteristics *viz*., protease (S1_VMI_ > S1)_7days_, amylase (S1_VMI_ > nS1)_3–7*days*_, and antioxidants (S1_VMI_ > S1)_7days_ levels. The partial least squares—discriminant analysis (PLS-DA) of gas chromatography—time of flight—mass spectrometry (GC-TOF-MS) datasets for primary metabolites exhibited a clustered pattern (PLS1, 22.04%; PLS2, 11.36%), with 7 days incubated S1_VMI_ extracts showed higher abundance of amino acids, sugars, and sugar alcohols with lower organic acids and fatty acids levels, relative to S1. Intriguingly, the higher amino acid and sugar alcohol levels were positively correlated with antioxidant activity, likely impeding SD in S1_VMI_. Further, the PLS-DA (PLS1, 18.11%; PLS2, 15.02%) based on liquid chromatography—mass spectrometry (LC-MS) datasets exhibited a notable disparity for post-SD (9–11 days) sample extracts with higher oxylipins and 13-desoxypaxilline levels in S1_VMI_ relative to S1, intertwining *Aspergillus* morphogenesis and secondary metabolism. The analysis of VOCs for the 7 days incubated samples displayed considerably higher accumulation of C-8 compounds in the headspace of twin-plate experimental sets (S1_VMI_:S2) compared to those in non-conditioned controls (S1 and S2—without respective partner strains), potentially triggering altered morpho-transformation and concurring biochemical as well as metabolic states in molds.

## Introduction

A variety of *Aspergillus* species have traditionally been employed for artisanal fermentation practices *viz*., making soy foods, seasonings, and beverages in Asian societies. Among the various mold varieties used in food fermentation, *A. oryzae* (trivially: koji mold) is the most revered species pertaining to its overwhelming secretion of hydrolytic enzymes (Sivaramakrishnan et al., 2007; Chancharoonpong et al., 2012). In general, *A. oryzae* (section *Flavi*) is supposedly a mitosporic mold under phylum Ascomycota (Subkingdom: Dikarya), though its potential mating types are also reported (Wada et al., 2012). Intriguingly, an *avant-garde* study by Wada et al. (2014) reports that *A. oryzae* can undergo heterokaryon formation, potentially generating sexual ascospores, inside the specialized structures known as sclerotia. Hence, these morphological transitions i.e., sclerotia development (SD), can further be linked with either the parasexual or likely sexual cycles in *A. oryzae* owing to its orthologous functions in closely related *A. flavus* (Horn et al., 2009). In addition, sclerotia are regarded as the surviving structures of molds to survive the exogenous stress conditions *viz*., insect fungivory (Gloer, 2007), oxidative stress (Grintzalis et al., 2014), or escaping host plant defense mechanisms during infection (Yang et al., 2016) etc. Notwithstanding its functional roles, the SD in mold species either trigger or co-regulates the production of various noble metabolites of pharmaceutical importance including penicillin and lovastatin, as well as deleterious mycotoxins (Calvo and Cary, 2015), in response to various exogenous factors and potentially the volatile organic compounds (VOCs) from ecotype species.

Either on natural or formulated growth microenvironments, particularly on solid-state growth matrices, the molds presumably establishes VOCs mediated interactions (VMI) with proximal microbial communities or host organisms. However, while the VOC trade-offs between fungi-prokaryotes (Schmidt et al., 2016, 2017) and fungi-plants (Lee S. et al., 2016; Cordovez et al., 2017) have been widely studied, the role of VOCs in fungi-fungi interactions are relatively unexplored. Particularly, the VMI between microbial communities involving molds, yeast, and prokaryotes in food matrices have attracted a great attention in contemporary foodomics. The artisanal practices for preparing the traditional fermented foods, i.e., *Koji* (steamed rice or wheat) or *Meju* (steamed and mashed soybean bricks), often involves *nuruk* (rice or barley straw) inoculation for transferring fermentative microflora. Since, a *nuruk* harbors an assortment of microbial communities including various strains of *A. oryzae* (Yang et al., 2013), we conjecture the likely spatiotemporal VMI between the ecotype strains on respective food matrices. Recently, we have shown that VMI between the two ecotype strains of *A. oryzae*, generally employed in food fermentation, selectively affects growth rates, primary metabolite profiles, and associated biochemical phenotypes (Singh and Lee, 2017). Hence, we assume that the effects of VMI on *Aspergillus* morpho-transformation i.e., SD, can be functionally correlated with associated biochemical properties (pH modulation, secreted enzymes, and antioxidants) as well as subtle metabolomes.

In this report, we propose a metabolomic yardstick to gauge the discriminant exometabolomes concomitant to SD in *A. oryzae* RIB 40 subjected to VMI with partner strain. In addition, we identified the putative headspace VOC's potentially engendering the observed morpho-transformation phenotypes. Mechanistically, the VOCs from partner strain appear to influence SD in proximally grown *A. oryzae* RIB 40 through regulating alterations in its primary and secondary metabolomes as well as the associated biochemical phenotypes. Taken together, this study demonstrates the role of VOCs as infochemicals vital for mold growth, metabolism, and perhaps in shaping the complex microbial community dynamics.

## Materials and methods

### Chemicals and reagents

All the chemicals and reagents used in the present study were of analytical grade. The fine chemical including acetonitrile (ACN), ethyl acetate (EA), dichloromethane (DCM), methanol (MeOH), and water were purchased from Fisher Scientific (Waltham, MA, USA). The standard oxylipins compounds were purchased from Cayman Chemical (Ann Arbor, MI, USA).

### Fungal strains, growth media composition, and culture conditions

The sclerotia forming *A. oryzae* RIB 40 (S1: KACC 44967) strain was procured from the Korean Agricultural Culture Collection (KACC), National Academy of Agricultural Sciences. Whereas, the partner strain of *A. oryzae* (S2: KCCM 60345) which doesn't undergo SD stages was supplied by the Korean Culture Center of Microorganisms (KCCM), The Republic of Korea. A variety of growth media were initially pre-screened for supporting SD in strain S1 *viz*., CZA (Czapek-Dox agar), WATM (Wickerman antibiotic test medium), SDA (Sabouraud dextrose agar), and MEA (Malt extract agar). The SD was evaluated through point inoculating each of the media with low density conidial suspension, 10 μL (1 × 10^6^ spores/mL) of fresh (14 days) pre-cultured conidia harvests from MEA (Brown et al., 2008). A modified low pH (~5) WATM agar medium with sucrose (2%) and low dextrose (0.1%) contents was used in the subsequent sets of experiments under standard laboratory conditions. The detailed composition of the modified WATM agar is provided as the Table S1. All cultures were incubated in dark at 28°C for 11 days, under standard laboratory conditions.

### Twin plate assembly for evaluating VOC's mediated interactions (VMI)

**Figure 1:**
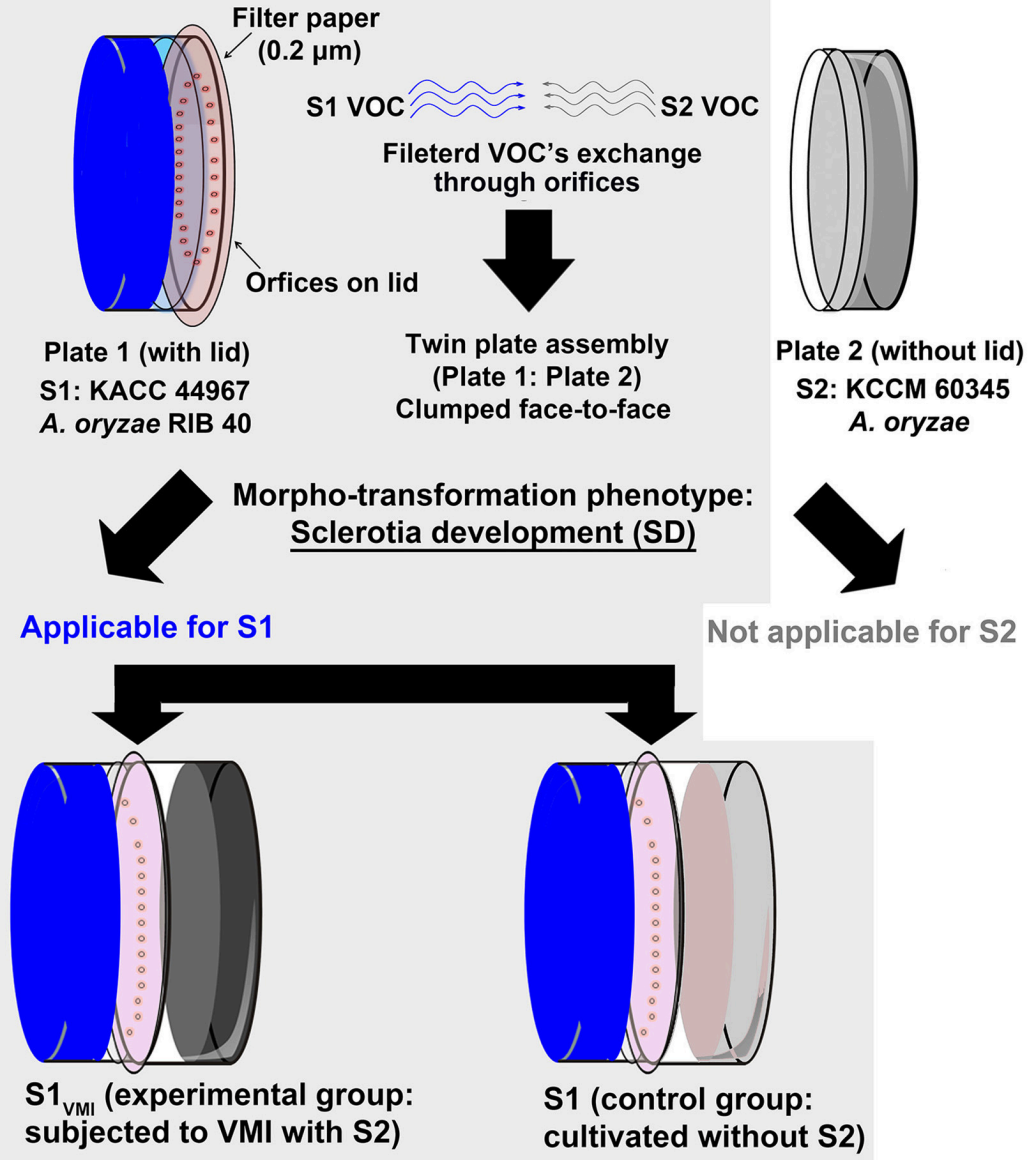
An illustrative diagram showing the twin plate assembly. The plate 1 with lid having orifice was inoculated with “strain 1” *Aspergillus oryzae* RIB 40 (KACC 44967), i.e., S1_VMI_ subjected to VOCs mediated interactions (VMI) with partner strain *A. oryzae* (S2: KCCM 60345) in plate 2. A control for strain 1 (S1) was maintained without partner strain (S2) in plate 2 of the twin plate assembly apparatus. All experimental procedures were performed for evaluating the effects of VMI in S1_VMI_ in comparison to its control S1 (marked with light gray shaded background), selectively in the context of morpho-transformation phenomenon (sclerotial development—SD) characteristic of strain 1 in the present study.

A twin plate assembly was engineered by coupling two petri dishes (P1 × P2) of regular sizes (100 mm × 15 mm), as described previously by Singh and Lee (2017). Here, the plate 1 (P1) was selectively inoculated with *A. oryzae* RIB 40 (designated as S1_VMI_: KACC 44967), with a lid having fixed number of fine orifice (25) for venting the VOCs exchange with partner strain of *A. oryzae* (designated S2: KCCM 60345) cultured in plate 2 (P2) without a lid. In order to avoid cross-contamination through orifice, the lid of P1 was externally covered with an autoclaved 0.2 μm filter paper matching the lid diameter. The twin plates (P1 × P2) were assembled opposite each other following the respective inoculations, and the rims were sealed with paraffin wax. The respective controls were also maintained, i.e., S1 (cultivated without the partner strain S2), in twin plate apparatus (Figure 1). Three biological replicates were maintained for each time point. Since, we were particularly interested in the effects of VMI on morpho-transformation phenomena i.e., SD, we focused on analyzing the growth, morphology, and associated biochemical and metabolomic alterations maneuvered in sclerotia forming *A. oryzae* RIB 40 (KACC 44967), comparing VMI conditioned S1_VMI_ and its control, S1. The strain S2 (*A. oryzae* KCCM 60345) having no characterized morpho-transformation phenotype was only considered as the source of VOCs, and only analyzed for its VOC contents.

### Sample harvest, growth profiling, and morphological examination of SD

*A. oryzae* RIB 40 samples (S1_VMI_ and S1) samples were harvested temporally at regular intervals; 0, 3, 5, 7, 9, and 11 days. The cultures were morphologically examined for SD and their growth radii (R) were measured to determine the associated radial growth rates (RGR).

### Sample preparation for pH evaluation and extraction

#### pH evaluation

Immediately following morphological examination, the harvested samples i.e., 1/3 of the biomass including agar (~8 g) from mashed biological replicates was subjected to pH measurement (Orion^TM^, 3 Star, Thermo Scientific).

#### Sample extractions for assaying biochemical phenotypes

Further, the biomass was used for crude enzyme extractions using the modified procedure partially adapted from Chancharoonpong et al. (2012). The mashed biomass (with agar) was agitated with an equal volume of 0.1 M phosphate buffer (pH 6.9) in an orbital shaker for 4 h at room temperature (26°C). The suspended biomass was centrifuged (13,000 × g, 10 min, 4°C) and the supernatant was collected as the source of crude enzyme.

#### Sample extractions for antioxidant assays and metabolic profiling

The sample extraction procedure for antioxidant assays (ABTS and DPPH) and metabolite profiling was adapted from Frisvad et al. (2014). The fungal biomass was first quenched using liquid N_2_, followed by subsequent pulverization with a pestle and mortar and mixer mill (frequency, 2,000/min). The pulverized biomass was extracted using a solvent mixture consisting of methanol: dichloromethane: ethyl acetate at (1:2:3) with formic acid (1%) and added chloramphenicol (25 μg/mL) as internal standard (IS). The pulverized samples were added to extraction solvent (1:1) and subjected to overnight agitation under incubating conditions (200 rpm, 28°C), followed by 1 h of ultra-sonication. The samples were centrifuged (5,000 × g) for 10 min at 4°C (Universal 320 R, Hettich, Zentrifugen, Germany) and the supernatants (200 μL) were collected into separate micro-centrifuge tubes. The sample supernatants were dried using speed vacuum sample concentrator (ModulSpin, Hanil Scientific, Korea). The dried samples were weighed and appropriate dilutions (10 mg/mL or 1,000 ppm) were made for subsequent analyses.

#### Sample extractions for VOCs analysis and cell concentration determination

The samples extraction methods for VOCs analysis was performed using the methods adapted from Costa et al. (2016). The fungal biomass (3 biological replicates) for *A. oryzae* RIB 40 (S1: KACC 44967) S1_VMI_ and S1 were harvested at stipulated time point by adding Ringer's solution (10 mL/per plate). The mycelial contents were separated from the agar and the harvested biomass was subjected to VOC profiling as well as simultaneous cell concentration determination (expressed as colony forming units, CFU/mL), maintaining 3 replicates for each dilution. The cell concentration data was used to normalize the VOCs profiling data for S1_VMI_ and S1. Linalool was used as internal standard (IS) for VOCs profiling.

### Biochemical phenotypes

#### Evaluation of enzymatic activities

The estimation of enzymes levels, i.e., proteases and amylases, in the crude extracts from fungal biomass (S1_VMI_ and S1) were made using the methods partially adapted from Bernfeld (1955), Kum et al. (2015), and Lee et al. (2017).

#### Assay for proteases (E.C: 3.4.21.19)

First, 1 mL of crude enzyme extract was mixed with 5 mL of casein solution (0.6% in 0.1 M phosphate buffer, pH 7), and then incubated for exactly 10 min at 37°C in a water bath. Immediately following incubation, the reaction was stopped by adding 5 mL of 0.4 M trichloroacetic acid (TCA) followed by a second incubation for 30 min at 37°C. The resulting precipitate in the reactions was filtered using a 0.2 μm PTFE (polytetrafluoroethylene) filter. Next, 5 mL of sodium carbonate (0.5 M) was added to adjust the pH drop prior to the addition of 1 mL of Folin's phenol reagents (0.5 M) to the filtrate (2 mL). The reactions mixture was again incubated for 30 min at 37°C, and its absorbance was recorded at 660 nm. A unit of protease was considered equivalent to the quantity of enzyme necessary to release 1 μg of tyrosine from 0.6% casein solution as a substrate in 1 min under standard conditions.

#### Assay for α-amylases (E.C: 3.2.1.1)

First, 1 mL of crude enzyme extract was mixed with 1 mL of starch solution (1% in 20 mM sodium phosphate buffer contaning 6.7 mM sodium chloride, pH 6.9), followed by 3 min incubation at 20°C. Next, 1 mL of color reagent (96 mM 3, 5-dinitrosalicylic acid solution in 5.3 M potassium sodium tartrate solution) was added to the reaction mixture and the solutions were incubated for 15 min in a boiling water bath. The samples were ice-cooled to room temperature and added with 9 mL of distilled water. The absorbance of the resulting solution was recorded at 540 nm. A unit of α-amylases was considered equivalent to the quantity of enzyme necessary to release 1 mg of free maltose from 1% soluble starch as the substrate solution in 1 min under standard conditions.

#### Evaluation of antioxidant activities

The estimation of antioxidant levels in the fungal biomass (S1_VMI_ and S1) were made using ABTS [2,2′-azinobis-(3-ethylbenzothiazoline-6-sulfonic acid)] and DPPH [2,2-diphenyl-1-picrylhydrazyl] based assays using the protocols partially adapted from Re et al. (1999) and Dietz et al. (2005), respectively. The methods are briefly described below;

#### DPPH assay

Each of the solvent extracts (20 μL) from fungal biomass i.e., 3 biological replicates (BR) × 3 analytical replicates (AR), was added upon with 0.2 mM DPPH solution in ethanol (180 μL) in a 96-well plate. The plate was incubated for 20 min in the dark at room temperature and absorbance was recorded at 515 nm.

#### ABTS assay

ABTS (7 mM) solution was prepared in potassium persulfate (2.45 mM) buffer, and the resulting solution was stored overnight at refrigerator (4°C). The solution was diluted with deionized water until its absorbance reached ~0.7 at 750 nm. Each of the solvent extract (10 μL) including 3BR and 3AR, were added upon with ABTS solution (190 μL) in 96-well plate. The reaction mixtures were incubated for 6 min in the dark at room temperature, and absorbance was immediately recorded at 750 nm using a microplate reader (Spectronic Genesys 6, Thermo Fisher, Madison, WI, USA).

### Metabolic profiling

#### GC-TOF-MS analysis for primary metabolites

##### Sample derivatization

First, the dried solvent extracts (adjusted for 1,000 ppm) were added with 50 μL of methoxyamine hydrochloride (20 mg/mL in pyridine) and the reaction mixture was incubated for 90 min at 30°C. Next, 50 μL of N-methyl-N-(trimethylsilyl) trifluoroacetamide (MSTFA) was added to the reaction, and the mixture was further incubated at 37°C for 30 min.

##### Instrumentation

The gas chromatography time-of-flight mass spectrometry (GC-TOF-MS) analysis was carried out using an Agilent 7890A GC system with an Agilent 7693 autosampler (Agilent, Santa Clara, CA, USA). The system was equipped with a Pegasus HT TOF-MS (Leco Corporation, St. Joseph, MI, USA). An RTx-5MS (fused silica) column with dimensions of 30 m length × 0.25 mm i.d. × 0.25 μm (J&W Scientific, Folsom, CA, USA), was used to separate derivatized metabolites with helium as carrier gas at a constant flow rate of 1.5 mL/min. The derivatized samples (1 μL) were injected into the GC-system under splitless mode. The injector and ion source temperatures were maintained at 250°C and 230°C, respectively, with column temperatures ramped from 75°C for the initial 2 min to 300°C at a rate of 15°C/min, and maintained for the final 3 min of the run cycle. All acquisitions were recorded at a fixed rate of 10 scans/s within a mass scan range of 50–500 m/z. Overall, the GC-TOF-MS analysis involved three BR, representing each time point of sample harvest. The analytical samples were analyzed in blocks of 10 runs followed by an intermittent QC (quality control sample, with 10 μL pooled blends from all samples) run. The analytical samples were randomized in each block.

##### Putative metabolite identification

The metabolites were putatively identified by comparing their retention time and mass fragment data with those retrieved from available databases *viz*., National Institute of Standards and Technology (NIST) database (version 2.0, 2011, FairCom, Gaithersburg, MD, USA) and our *in-house* library (~6500 metabolites) of standard compounds.

#### LC-MS analyses for secondary metabolites

##### Instrumentation

The ultra-high-performance liquid chromatography linear trap quadrupole ion trap tandem mass spectrometry (UHPLC-LTQ-IT-MS/MS) system was equipped with an LTQ ion trap MS, operated using Xcalibur^TM^ v2.2 software (Thermo Fisher Scientific Inc., San José, CA). The rapid separation (RS) chromatographic system was equipped with a binary solvent delivery system with an RS autosampler (Thermo Fishcher Scientific Inc.) and RS Pump (DIONEX UltiMate 3000, Sunnyvale, CA, USA), and RS Diode Array Detector (Dionex Corporation). The samples were separated on a Syncronis C18 UHPLC column with dimensions, 100 mm × 2.1 mm, 1.7 μm particle size (Thermo Scientific), using the mobile phase consisting of water (solvent A with 0.1% HCOOH, v/v) and acetonitrile (solvent B with 0.1% HCOOH, v/v). The mobile phase run program was; 10% solvent B for 1 min, ramped to 100% solvent B over 18 min, kept constant for the next 3 min, and a finally re-establishment to the initial conditions (10% solvent B) within 1 min. The sample injection volume and flow rates of mobile phase were maintained at 10 μL and 0.3 mL/min, respectively. The photodiode array (PDA) and mass spectrometry (MS) detectors in both of positive and negative ion modes were tuned for wavelength range 200–600 nm and m/z range, 100–1,000, respectively, under full scan positive and negative ion modes. The system parameters *viz*., capillary temperature, voltage, and source voltage were tuned for 275°C, 39 V, and ± 5 kV, respectively. The analysis was performed for 3 biological replicates (BR), representing each time point involving sample harvest. The analytical samples were randomly analyzed in blocks of 10 runs followed by an intermittent QC (quality control sample, with 10 μL pooled blends from all samples) run to ascertain the instrumental drift.

Further, ultraperformance liquid chromatography (UPLC) ACQUITY system (Water Corp., Milford, MA, USA) was interfaced with a micro mass quadrupole—time of flight—mass spectrometry (Q-TOF) premier system (Micromass MS technologies, Manchester, UK), operated using the Masslynx v4.1 software. The metabolite separation was performed on an ACQUITY BEH C18 column with dimensions 100 mm × 2.1 mm, 1.7 μm particle size (Waters Corp.) at a constant flow rate of mobile phase (solvent A-−0.1% v/v, HCOOH in water; solvent B-−0.1% v/v, HCOOH in acetonitrile) at 0.3 mL/min, and with a sample injection volume of 5 μL. The chromatographic run program was configured as follows: 5% solvent B for the initial 1 min, followed by a gradient increased to 100% of solvent B over 9 min, maintaining at 100% solvent B for the next 1 min, and final re-equilibration of the column to initial conditions i.e., 5% of solvent B over the next 3 min. The total run program was of 14 min. We performed an untargeted metabolite profiling with electrospray ionization (ESI) of analytes in both negative and positive ion modes covering a mass range of 100–1,000 m/z. The capillary and cone voltages were tuned for 2.5 kV and 30 V, respectively. The dissolving gas flow rate was set to 700 L/h at a temperature of 300°C. The collision energy was 5 eV, with a source temperature of 100°C.

##### Putative metabolite identification

The metabolites evaluated by UPLC-LTQ-IT-MS/MS were tentatively identified through comparing their molecular weights (M.W), retention times (RT), mass fragmentation patterns (MS^n^) in tandem MS spectrometry, and UV absorbance data with those obtained from published literatures and our *in-house* library, and databases. Further, the elemental compositions and molecular formulae for the candidate metabolites were examined using the UPLC-Q-TOF data with low mass errors, i.e., ≤5 ppm.

#### HS-SPME-GC-TOF-MS analysis for VOC's

##### VOCs extraction

The Ringer's solution harvests of S1_VMI_ and S1 samples were centrifuged (13,000 × g, 10 min, 4°C) and the supernatants (10 mL) were transferred to SPME glass vials (20 mL), followed by the addition of 2 g of NaCl (analytical grade, Sigma Aldrich). The headspace - solid phase microextraction (HS-SPME) of the VOCs was performed using divinylbenzene/carboxen™/polydimethylsiloxane (DVB-CAR-PDMS) StableFlex™ (1 cm) fiber (Sigma-Aldrich). The VOC's extraction was performed by exposing the SPME fiber to the headspace of sample supernatants for 30 min at 50°C under a thermostat water bath. At the end of SPME procedure, the extraction fiber was removed with SPME fiber holder and desorbed at the GC port (270°C) for 5 min.

##### GC-TOF-MS analysis of VOCs

The samples were analyzed according to the methods described previously by Singh and Lee (2017).

##### Putative VOCs identification

The metabolites were identified through comparing their RT and mass fragment data (MS) with the data retrieved from National Institute of Standards and Technology (NIST) database (FairCom, USA; ver. 2.0, 2011), Wiley Registry of Mass Spectral Data (9th Ed.), and VocBinBase (Skogerson et al., 2011).

### Data processing

The data files obtained from GC-TOF-MS and UHPLC-LTQ-IT-MS/MS analyses were converted to netCDF (^*^.cdf) format using ChromaTOF software (LECO corp.) and Xcalibur software (version 2.00, Thermo Fisher Scientific Inc., San José, CA), respectively. The obtained (^*^.cdf) data files were subjected to data pre-processing for retention time, normalized peak intensities, and accurate masses using MetAlign software package (http://www.metalign.nl), and the alignment data were exported to Excel format. The multivariate statistical analysis for aligned metabolic profiling data was carried out using SIMCA-P+ (version 12.0, Umetrics, Umea, Sweden). The principal component analysis (PCA) and partial least squares—discriminant analysis (PLS-DA) were performed to identify the class-wise variance in the datasets. The significantly discriminant metabolites among the temporal metabolomic datasets of S1_VMI_ and S1, were selected based on the variable importance in the projection values at VIP > 0.7 and *p* < 0.05. Further, orthogonal projection to latent structures discriminant analysis (OPLS-DA) was performed to evaluate the categorical disparity in selected datasets. For biochemical phenotypes *viz*., protease, amylase, ABTS, and DPPH assays, the pair-wise comparisons were made using analysis of variance (ANOVA) and Duncan's multiple range tests, in PASW statistica 18 software (SPSS Inc., Chicago, IL, USA). The heat map based on processed and aligned GC-TOF-MS data was visualized using MeV software (v 4.8, multiple array viewers, TM4), followed by Pearson's pair-wise correlation analysis between the levels of metabolites and selected phenotypes in PASW statistica 18.

## Results

### Effects of VMI on SD and mycelial growth as well as concomitant modulation of media pH and biochemical phenotypes

**Figure 2:**
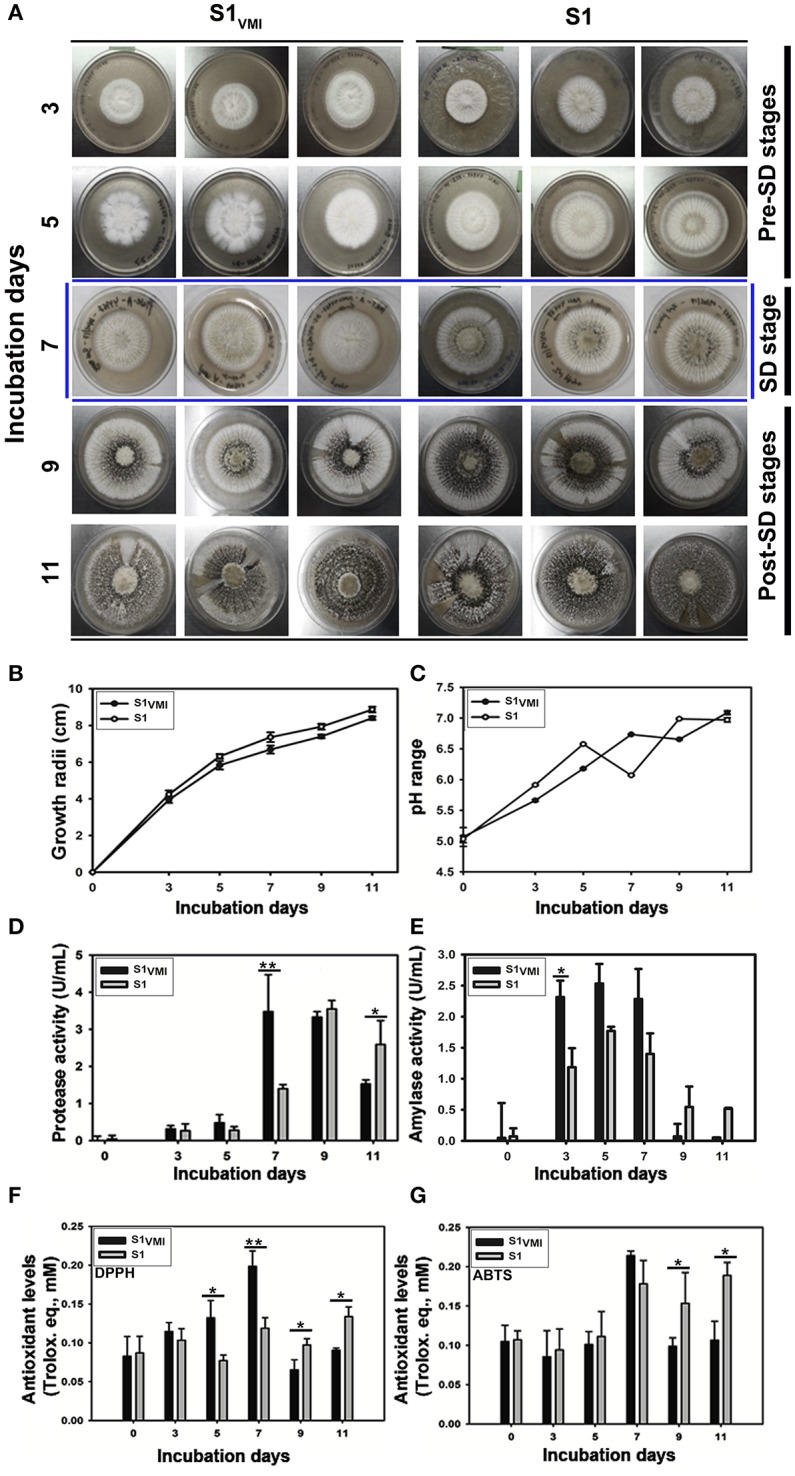
A time-correlated comparative evaluation of **(A)** culture morphology and SD, **(B)** growth profiles, **(C)** media pH variations, **(D)** protease activity, **(E)** Amylase activity, **(F)** DPPH antioxidant assay, **(G)** ABTS antioxidant assay, for the sclerotia forming *A. oryzae* RIB 40 (S1_VMI_ and S1). Here, the data represent the mean ± SD of the triplicates representing three biological sets for each sample (S1_VMI_ and S1). The asterisk representation for one-way ANOVA Dunkan-Tukey pair-wise comparison test indicates, ^**^p < 0.01, ^*^p < 0.05.

The twin plate assembly engendered the potential VMI between the two ecotype strains of *A. oryzae* (S1: KACC 44967 and S2: KCCM 60345), however only the sclerotia forming strain 1, i.e., S1_VMI_ (conditioned group: subjected to VMI with S2) and S1 (control group: cultivated without S2), with characteristic morpho-transformation phenotype, SD, were selectively examined. We observed a marked delay of 2 days in SD for S1_VMI_ (9 days) compared to control strain S1 (7 days), incubated in twin plate assembly (Figure 2A). As shown in Figure 2B, the delay in SD for S1_VMI_ was also accompanied by relatively slower RGR compared to S1. Considering the RGR data for 7 days incubated samples, S1_VMI_ and S1 exhibited the average growth rates (area occupied by mycelial growth/incubation days) of 4.98 ± 0.34 and 6.09 ± 0.50 cm^2^/day, respectively. Intriguingly, lowered pH values of the cultivation media were recorded on 7th day for S1 and 9th day for S1_VMI_ synchronous to their SD stages (Figure 2C). Earlier, Rollins and Dickman (2001) have described the low pH conditions as conducive toward promoting the SD in certain mold species.

Analyzing the biochemical phenotypes, we observed a significant disparity between 7 days incubated samples characterized morphologically with predominant mycelial forms in S1_VMI_ and the SD in S1 (marked with blue colored boundaries, Figure 2A). In accordance to the morphological disparity, the levels of extracellular proteases between 7 days incubated S1_VMI_ were significantly higher than corresponding S1 samples, *p* < 0.01. Intriguingly, during the post-SD stages of incubation (9 and 11 days), the protease levels in S1_VMI_ were decreased following the SD, with significantly higher protease levels in S1 relative to S1_VMI_ at 11 days, *p* < 0.05 (Figure 2D). On the other hand, the amylase activity for S1_VMI_ samples was relatively higher than S1 up to 7 days, followed by a sharp decrease during post-SD stages for both the groups (Figure 2E). We further observed concordant trends for antioxidant levels with 5 days and 7 days incubated S1_VMI_ samples, which exhibited significantly higher DPPH radical scavenging activities than S1, at *p* < 0.05 and *p* < 0.01, respectively (Figure 2F). Conversely, during the post-SD stages the antioxidant levels in S1 were relatively higher (*p* < 0.05). Although, the antioxidant levels determined using ABTS assay showed marginal differences between S1_VMI_ and S1, and the trends of antioxidant levels were similar to those determined using the DPPH assay (Figure 2G).

### Effects of VMI on exometabolomes

#### Primary metabolomes

**Figure 3:**
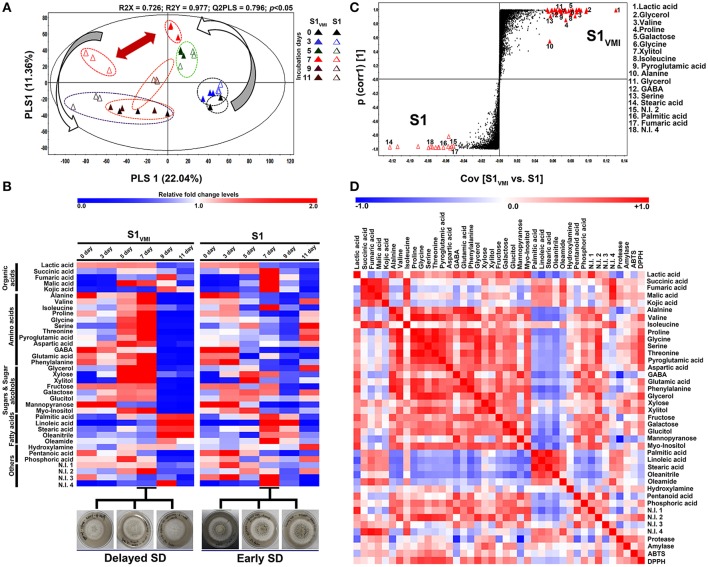
A time-correlated **(A)** PLS-DA score plot displaying the clustering patterns of metabolomic datasets, **(B)** heat map representation for the relative fold change levels of significantly discriminant metabolites (VIP > 0.7, *p* < 0.05), **(C)** S-plot for OPLS-DA analysis showing the discriminant metabolite biomarkers (VIP > 6, *p* < 0.05) for 7 days incubated samples, and **(D)** the heat map showing Pearson's correlation for linearity between detected metabolite patterns and associated biochemical phenotypes, derived from GC-TOF-MS detected primary metabolite datasets for *A. oryzae* RIB 40 (S1_VMI_ and S1) sample extracts.

The GC-TOF-MS detected metabolomic datasets mainly included primary metabolites showing a temporal propensity and associated disparity between S1_VMI_ and S1. The PCA (Figure S1A) and PLS-DA (Figure 3A) datasets exhibited an overall variability of 35.01% (PC1: 23.42%, PC2: 11.59%) and 33.4% (PLS1: 22.04%, PLS2: 11.36%), respectively. The PLS-DA score plot revealed a clear disparity in the metabolomic datasets from 7 days incubated S1_VMI_ and S1 along PLS1 (22.04%), which was concomitant to the disparity in their morphological states, i.e., mycelia in S1_VMI_ and sclerotia in S1. The significance of PLS-DA models were verified with R^2^X (0.726), R^2^Y (0.977), and Q^2^ (0.796) parameters, indicating the fitness and prediction accuracy of model at *p*-value (<0.05) obtained through cross-validation (Figure 3A). Considering the time-resolved metabolomes for all incubations days, we identified 37 significantly discriminant metabolites (VIP > 0.7 and *p* < 0.05), including organic acids (5), amino acids (12), sugar and sugar alcohols (8), fatty acids and their derivatives (5), organic compounds (3), and the non-identified compounds (4) as shown in Table S2. The heat map representation for the relative abundance of significantly discriminant metabolites showed a marked disparity for the 7 days incubated samples, followed by those harvested at 5 and 11 days (Figure 3B). The 7 days incubated S1_VMI_ sample extracts were characterized with higher relative abundance of amino acids, sugars, and sugar alcohols. On the other hand, a relatively higher abundance of organic acids (except lactic acid), fatty acids, and fatty acid derivatives were observed for S1. Similar trends for metabolite levels were observed for 5 days incubated samples, while the samples harvested at 11th day exhibited a comparatively lower disparity involving mainly the amino acids and fatty acids.

Selectively, the GC-TOF-MS datasets for 7 days incubated sample extracts were subjected to OPLS-DA analysis (Figure S1B), while the metabolite biomarkers discriminating S1_VMI_ and S1 were determined from S-plot, VIP > 6.0 and *p* < 0.05, represented at the extreme opposite ends of the plot (Figure 3C). The S-plot analysis indicated that most amino acid (valine, proline, glycine, isoleucine, pyroglutamic acid, alanine, GABA, serine), sugar and sugar alcohols (glycerol, galactose, xylitol, glycerol), and lactic were associated with predominant mycelial forms in S1_VMI_ samples. In contrast, fatty acids (stearic acid, palmitic acid) and fumaric acid were associated with sclerotial forms in S1 samples. However, most biomarker metabolites remained unidentified, indicated with large sized but unassigned points in the S-plot (Figure 3C).

To examine the linear correlations between the GC-TOF-MS detected significantly discriminant primary metabolites and biochemical phenotypes (protease, amylase, and anti-oxidant assays), we performed the Pearson correlation analysis (*p* < 0.05). Intriguingly, a strong positive correlation was observed between DPPH antioxidant activity and most of the amino acids (valine, proline, glycine, serine, threonine, pyroglutamic acid, aspartic acid, and glutamic acid) as well as sugar alcohols (glycerol and xylitol). Similar but relatively weaker correlations were observed between these metabolite sets and ABTS antioxidant assays (Figure 3D).

#### Secondary metabolomes

**Figure 4:**
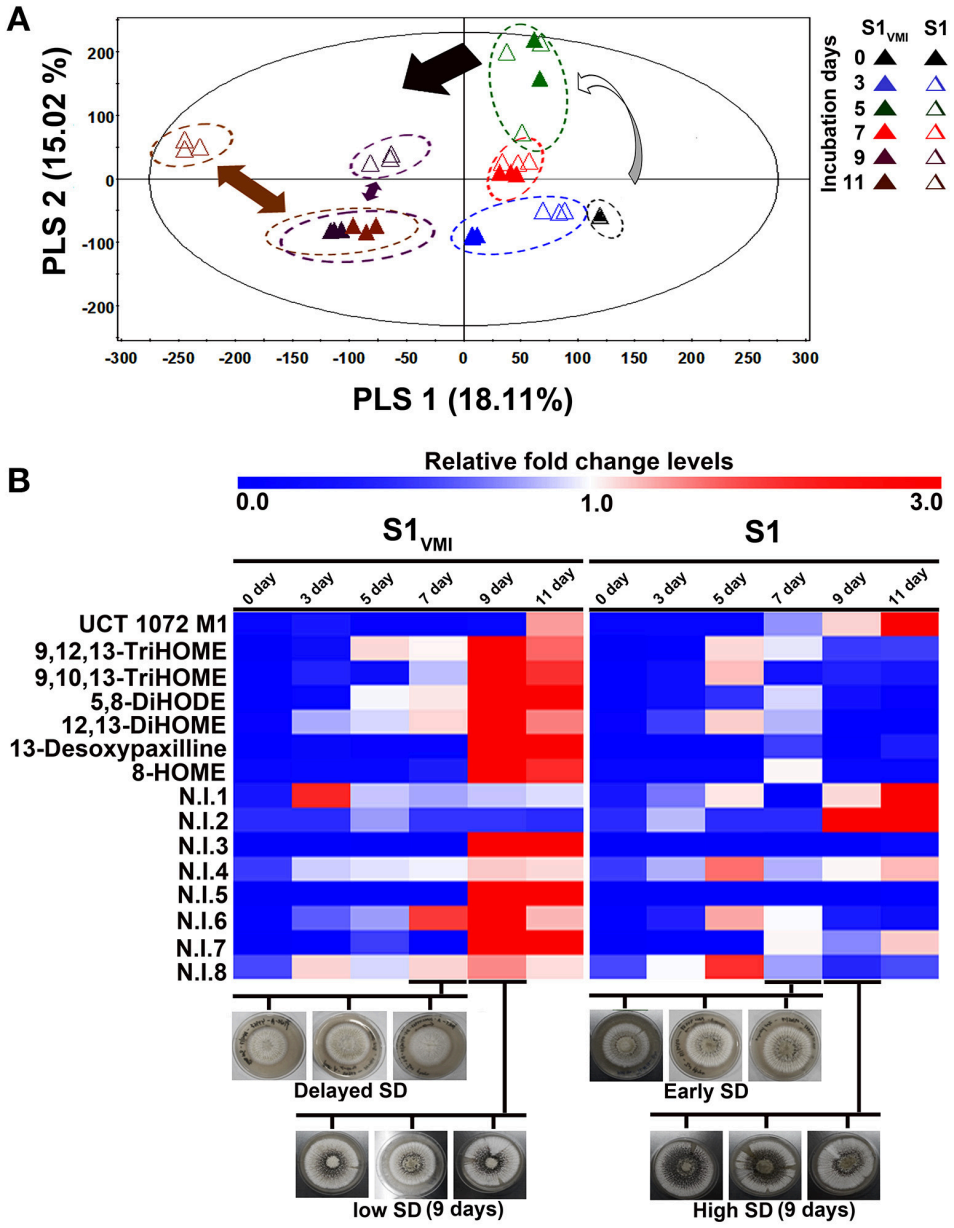
A time-correlated **(A)** PLS-DA score plot displaying the clustering patterns of metabolite, and **(B)** the heat map representation for the relative fold change levels of significantly discriminant metabolites (VIP > 0.7, *p* < 0.05), derived from UHPLC-LTQ-IT-MS/MS detected secondary metabolite datasets for *A. oryzae* RIB 40 (S1_VMI_ and S1) sample extracts.

The LC-MS examined datasets mainly included secondary metabolites. Notably, the temporal multivariate analyses based on UHPLC-LTQ-IT-MS/MS datasets for negative (-electrospray ionization, ESI) mode displayed a marked disparity for 9D and 11D incubated sample extracts, incongruent to the trends observed for primary metabolites with higher disparity for 7 days incubated samples. The PCA (Figure S2) and PLS-DA (Figure 4A) score plots exhibited a total variability of 40.93% (PC1: 24.66%, PC2: 16.27%) and 33.13% (PLS1: 18.11%, PLS2: 15.02%), respectively. The secondary metabolites representing the post SD stages i.e., 9 and 11 days incubated S1_VMI_ and S1 were clearly demarcated along PLS2 (15.02%). The PLS-DA model validation was performed through determining the R^2^X (0.679), R^2^Y (0.993), and Q^2^ (0.882) parameters.

Further, we putatively identified the significantly discriminant secondary metabolites associated with the time-correlated morphological transformation, i.e., SD in S1_VMI_ and S1, using PLS-DA model (VIP > 0.7 and *p* < 0.05). The putatively identified metabolites with characteristic chromatographic profiles and mass spectral properties are listed in Table 1. Among the selected discriminant metabolites, we mainly identified the oxygenated polyunsaturated fatty acid, i.e., oxylipin compounds. Additionally, an anthraquinone furan derivative (UCT 1072M1), an indole diterpene (13-desoxypaxilline) compound, and eight non-identified (N.I) metabolites were characterized as significantly discriminant between S1_VMI_ and S1. The relative levels of these significantly discriminant metabolites were conspicuously distinct for the post SD-stages S1_VMI_ and S1 sample extracts (Figure 4B). In particular, a reciprocal relationship has been proposed for the extracellular levels of oxylipin compounds and conidiation stages, promoting the SD in *Aspergillus* species (Brown et al., 2008).

**Table 1:** The list of putatively identified significantly discriminant metabolites (VIP > 0.7, *p* < 0.05) based on PLS-DA datasets obtained through the temporal LC-MS analyses of *A. oryzae* RIB 40 (S1_VMI_ and S1) sample extracts.

	**UHPLC-LTQ-IT-MS/MS**							**UPLC-Q-TOF-MS**					**ID/References**
**S. no**.	**RT (min)**	**Tentatively identified metabolites**	**[M-H]^−^**	**[M-H]^+^**	**M.W**	**MS^n^ fragments**	**λ _max_ (nm)**	**Measured [M-H]^−^**	**Mol. form**.	**iFi*t***	**PPM**	**Error (mDa)**	
1	9.42	UCT 1072M1 (Anthraquinone furan derivative)	355	357	356	(–)355 > 311 > 293, 283, 269, 268, 266 > 249; (+)357 > 339 > 297 > 269, 255, 241, 213, 122	214, 220, 292, 436	355.0454	C18H11O8	0.7	0.8	0.3	Asai et al., 1999
2	11.22	9,12,13-Trihydroxy-10-octadecenoic acid (9,12,13-TriHOME)	329	331	330	(–)329 > 311 > 293, 275, 181,155; (+)331 > 314, 295, 226, 200, 104	219, 228, 274	329.2328	C18H33O5	0.1	−2.1	−0.7	Lee et al., 2016a
3	11.30	9,10,13-Trihydroxy-10-octadecenoic acid (9,10,13-TriHOME)	329	331	330	(–)329 > 311 > 293 > 275, 249, 193	219, 227	329.2321	C18H33O5	0.1	−2.1	−0.7	Martin-Arjol et al., 2010
4	11.91	5,8-Dihydroxy-octadeca-9,12-dienoic acid. (5,8-DiHODE)	311	–	312	(–)311 > 293 > 275, 249 > 231, 193, 181, 165, 149;	221	311.2222	C18H31O4	0.5	0.0	0.0	Garscha and Oliw, 2007
5	13.42	12, 13-Dihydroxy-octadeca-9-monoenoic acid. (12,13-DiHOME)	313	315	314	(–)313 > 295, 276, 201 > 277, 251, 195, 179, 171; (+)315 > 297, 279 > 277, 251 > 195, 179 > 261, 243	222, 231	313.2379	C18H33O4	0.2	−3.5	0.2	Standard compound
6	16.53	13-Desoxypaxilline (Indole diterpene)	464^f^	420	419	(+)420 > 402 > 384, 330, 285, 238 > 182,167	233, 274, 487	418.2382	C27H32NO3	0.0	−1.9	−0.8	Rank et al., 2012
7	18.55	8-Hydroxy-octadeca-9-monoenoic acid, (8-HOME) Psi B - β	297	–	298	(–)297 > 279, 254, 253 > 249, 225	225	297.2430	C18H33O3	0.0	−5.4	−1.6	Smith et al., 2005 (Metlin DB)
**NON-IDENTIFIED METABOLITES**													
8	8.82	N.I (1)	321,367 ^f^	–	322	(–)367 > 321 > 257 > 221,194	213, 273	–	–	–	–	–	–
9	9.05	N.I (2)	199	–	200	(–)199 > 181,155, 137, 127	213, 269, 320	–	–	–	–	–	–
10	9.55	N.I (3)	373	–	374	(–)373 > 355, 315 > 313, 287 > 259, 243, 215;	215, 291, 366	373.0560	–	–	–	–	–
11	10.92	N.I (4)	464	466	465	(–)464 > 402, 420, 446 > 398, 382, 353 > 366, 351, 314, 298 (+)466 > 448, 430, 412, 355	219, 226, 291	464.2473	–	–	–	–	–
12	11.10	N.I (5)	357	–	358	(–)357 > 339, 299 > 339, 321, 297 > 253	219, 277, 288	357.0610	–	–	–	–	–
13	11.59	N.I (6)	329, 659	331, 661	330	(–)659 > 329 (+)661 > 642, 643	220	329.0661	–	–	–	–	–
14	12.38	N.I (7)	339	341	340	(–)339 > 297, 321, 339 > 293, 275, 231, 187	221, 230	339.0505	–	–	–	–	–
15	12.57	N.I (8)	448	450	449	(–)448 > 404 (+)450 > 432, 414	222	–	–	–	–	–	–

*RT, Retention time for chromatographic elution; f: [M+FA–H], where FA: Formate adduct; S1_VMI_, Strain 1 (A. oryzae RIB 40, KACC 44967) subjected to VOC's mediated interaction with partner strain, S2 (A. oryzae, KCCM 60345) in twin plate assembly; S1, Strain 1 (A. oryzae RIB 40, KACC 44967) cultivated without partner strain in twin plate assembly*.

### The VOC's in the common headspace of S1_VMI_ and S2

We observed a notable disparity in the duration of morpho-transformation phenotypes (SD) for 7 days incubated S1_VMI_ and S1, with concomitantly distinct biochemical and metabolic characteristics. Hence, we analyzed the headspace VOCs potentially affecting the SD in 7 days incubated S1_VMI_ subjected to VMI with partner strain S2. The details of chromatographic as well as mass spectrometric characteristic of the putatively identified VOCs are provided in Table 2. Intriguingly, a notably higher abundance of C-8 VOCs *viz*., 1-octen-3-ol, (5Z)-octa-1,5-dien-3-ol, 2-octenal, and 3-octanone was found associated with experimental groups S1_VMI_ conditioned for VOC trade-offs with partner strain S2 in twin-plate assembly, corroborating our previously published study (Singh and Lee, 2017). However, we also observed a diverse range of VOCs including aldehydes, alcohols, aryl alkenes/aryl aldehydes, and ketones, with distinct compounds in comparison to our previous report, owing to the different methods employed.

**Table 2:** HS-SPME-GC-TOF-MS analysis of putatively identified headspace VOCs for 7 days incubated experimental (S1_VMI_:S2) and respective control (S1 and S2), strains of *A. oryzae*.

**S. no**.	**RT ± SD (min)**	**Identified VOCs**	**Fragment ion intensity**	**UM**	**Peak area** ± **S.D (Normalized with CFU and Internal standard)**			**ID source**	**Chemical class**
					**S1_VMI_:S2**	**S1**	**S2**		
1	1.28 ± 0.14	Hexanal	44, 56, 41, 57, 43, 39, 72	44	N.D	N.D	0.0076 ± 0.003	NIST	Aldehyde
2	1.31 ± 0.15	N.I. 1	44, 207, 96, 208, 209, 191, 133	207	0.04 ± 0.0085	N.D	N.D	N.D	N.A
3	4.84 ± 0.57	Ethenyl benzene	104, 78, 103, 77, 51, 44, 52	104	N.D	N.D	0.0011 ± 0.0008	Wiley 9	Aryl alkenes
4	10.41 ± 0.31	Benzaldehyde	77, 105, 106, 51, 50, 78 52	77	N.D	N.D	0.018 ± 0.0022	NIST	Aldehyde
5	11.37 ± 0.19	(5Z)-Octa-1,5-dien-3-ol	57, 55, 41,70, 39, 42, 69	57	0.29 ± 0.11	0.0043 ± 0.0013	0.063 ± 0.004	Wiley 9	Alcohol
6	11.71 ± 0.10	1-Octen-3-ol	57, 43, 72, 41, 55, 39, 58	57	15.38 ± 7.15	0.54 ± 0.18	4.02 ± 0.0054	Wiley 9 VOC BinBase	Alcohol
7	11.93 ± 0.014	3-Octanone	57, 43, 72, 99, 41, 71, 55	99	0.046 ± 0.032	N.D	0.024 ± 0.0045	NIST	Ketone
8	12.10 ± 0.19	7-methyl-3-methylideneocta-1,6-diene	41, 93, 69, 57, 91, 39, 77	93	N.D	0.0054 ± 0.0018	0.003 ± 0.00022	NIST/VOC BinBase	Alkene
9	12.34 ± 0.50	N.I. 2	57, 41, 43, 93, 69, 55, 39	57	N.D	N.D	0.000544 ± 0.0001	N.D	N.A
10	12.58 ± 0.16	Octanal	44, 57, 41, 43, 55, 56, 42	56	N.D	N.D	0.0051 ± 0.00054	Wiley 9	Aldehyde
11	12.67 ± 0.077	N.I. 3	281, 282, 283, 265, 133, 249, 73	281	0.14 ± 0.0037	0.0043 ± 0.0018	N.D	N.D	N.A
12	13.98 ± 0.09	Benzene acetaldehyde	91, 92, 65, 120, 39, 51, 63	91	N.D	N.D	0.020 ± 0.017	Wiley 9	Aryl aldehyde
13	14.57 ± 0.041	2-octenal	41, 55, 70, 57, 39, 83, 42	55	0.23 ± 0.035	0.022 ± 0.011	0.015 ± 0.0021	Wiley 9	Aldehyde
14	15.17 ± 0.10	1-Octanol	56, 55, 41, 43, 69, 42, 70	56	N.D	N.D	0.070 ± 0.0082	NIST	Alcohol
15	16.42 ± 0.095	Linalool	71, 43, 93, 41, 55, 69, 80	71	1.016 ± 0.022	1.0 ± 2.54 × 10^−7^	1.0 ± 1.59 × 10^−7^	IS	Terpene alcohol
16	17.14 ± 0.14	1-octen-3-yl acetate	43, 54, 99, 67, 41, 39, 55	99	0.0043 ± 0.00082	0.0034 ± 0.00037	N.D	NIST	Ester

*RT, Retention time; VOCs, Volatile organic compounds; UM, Unique mass; N.I, Not identified; N.D, Not determined; NIST, National Institute of Standards and Technology; Wiley 9, Wiley Registry of Mass Spectral Data, 9th edition; S1_VMI_:S2, Peak area for the VOC's shared between the common headspace of A. oryzae RIB 40 (S1_VMI_: KACC 44967) and partner strain A. oryzae (S2: KCCM 60345). S1, Peak area for the VOC's from the headspace of A. oryzae RIB 40 (S1: KACC 44967). S2, Peak area for the VOC's from the headspace of A. oryzae (S2: KCCM 60345)*.

## Discussion

Considering the promiscuous colonization patterns of molds in the natural environment or formulated semi-natural matrices, facile but incontrovertible interactions among the proximal species are indispensable. Provided the unregulated or low water activity conditions in fungal niches *viz*., soil, agricultural commodities, hosts microenvironment, or *in situ* solid state cultivation etc., the contactless VMI might have categorically shaped fungal ecology. Hence, VMI are increasingly been recognized as the “*terra incognita”* of microbe—microbe interactions among the ecotype strains occupying a common niche. Functionally, the VMI are discussed and studied under the domains of fundamental ecological interactions *viz*., antagonistic, ammensalic, or commensalic, potentially modulating the metabolic pathways and characteristic phenotypes among interacting species (Chatterjee et al., 2016; Schmidt et al., 2017). In the present study, we aimed primarily to probe the effects of intra-species VMI on *A. oryzae* RIB 40 (strain S1: KACC 44967) cultured in close proximity opposite the partner strain, *A. oryzae* (S2: KCCM 60345).

The morphological transformation, i.e., SD in *Aspergillus* species is considered vital for their survival, as the sclerotia are overwintering structures enabling cells to withstand environmental stress conditions such as oxidative and pH stress (Grintzalis et al., 2014; Xu et al., 2015). Firstly, we designed a low pH (~5) modified WATM agar medium conducive for SD in *A. oryzae* RIB 40 (S1: KACC 44967). Hence, it was conceived that SD in S1 would serve as an important phenotype for comparatively evaluating the effects of VMI on SD and exometabolomes in S1_VMI_ cultivated proximally opposite the partner strain S2, in twin plate assembly (Figure 1). We surmise that the proposed twin-plate experiment further corroborated as well as complemented the previously described experimental setups for evaluating the VMI among microbes *viz*., plate-within-a plate (Schmidt et al., 2017) and multi-well plates assembly (Cernava et al., 2015).

In our experiment, a direct exposure of S1_VMI_ to the filtered VOCs emanated from partner strain (S2) delayed the morpho-transformation of mycelia to sclerotia in the former relative to the corresponding control S1, which can be attributed to the abstruse intra-species signaling between the two strains. We assume that the growth, morphological transformation, and metabolism in molds are tightly regulated and intertwined resisting exogenous perturbations including VOC's. In the low pH (~5) WATM agar medium conducive for SD, we observed an initial temporal rise in pH levels for both S1_VMI_ and S1, followed by a sharp pH fall concurrent to SD following 7 and 9 days of incubation, respectively. Reportedly, the low pH extracellular environment favors SD in some mold species (Rollins and Dickman, 2001). Hence, a delayed pH fall in S1_VMI_ might be associated to its delayed SD and vice versa. We conjecture that higher extracellular abundance of organic acids might have maneuvered the low pH environment essential for SD. In accordance, the chronology of organic acid abundance was observed concomitant to the SD in S1 (7th day) and S1_VMI_ (9th day), at SD and post-SD stages, respectively (Figure 3B). Previously, it has been argued that extracellular pH and organic acid production in *Aspergillus* species are linked to pal/pacC pH signaling pathway (Andersen et al., 2009), regulating its metabolism and morpho-transformation (Rollins, 2003). A sharp disparity in the relative levels of organic acids was noteworthy between the S1_VMI_ and S1 sample extracts following 7 days of incubation, with lactic acid and fumaric acid were identified as the biomarker primary metabolites, respectively.

The relatively higher levels of secreted hydrolytic enzymes, proteases and amylases, in S1_VMI_ during SD and SD/post-SD stages, respectively, might have maneuvered the primary metabolomes *viz*., extracellular amino acids, sugars, and sugar alcohols. Further, we observed that the temporal disparity in the levels of primary metabolites significantly influenced the oxidative states of incubated strains concurrent to SD stages in S1_VMI_ and S1 (Figures 2D–G). It was reported decades back that free amino acids, especially S-containing, in extracellular environment or local niche of certain mold species either inhibit or delay SD (Trevethick and Cooke, 1971; Moromizato et al., 1980). In our study, following the 7 days of incubation S1_VMI_ samples with predominant mycelial forms showed the higher relative abundance of free amino acids, identified as biomarkers metabolites (Figures 3B,C). Though, we didn't identify any S-containing amino acids, the overall higher abundance of free amino acids (except isoleucine) in 7 days incubated S1_VMI_ extracts could have potentially eventuated into an anti-oxidative milieu, delaying SD. Reportedly, the elevated oxidative stress is assumed as the main abiotic factor triggering the SD in *Aspergillus* species (Grintzalis et al., 2014). The antioxidant potentials of free amino acids are widely corroborated under the perspectives of food and dietary supplements (Duan et al., 2016; Lee et al., 2016b). Notwithstanding the amino acids, the relative levels of sugar alcohols *viz*., glycerol, xylitol, glucitol, and myo-inositol were conspicuously higher in 7 days incubated S1_VMI_ extracts (Figure 3B). Similar to amino acids, the antioxidant activities of sugar alcohols can also be attributed to the delayed SD in S1_VMI_. Sugar alcohols are reported for free radical scavenging activities alleviating oxidative stress in certain plant and yeast species (Keunen et al., 2013; Meena et al., 2015). The results were statistically verified through visualizing the strong positive correlations among antioxidant phenotypes, amino acids, and sugar alcohol levels (Figure 3D).

A seminal study describing SD in *Aspergillus* species have illuminated the pivotal importance of free fatty acids, especially poly-unsaturated fatty acids (PUFA), toward SD (Calvo et al., 1999). The authors have meticulously described a linear relation between SD and linoleic acid doses in *A. flavus* incubated under dark conditions. However, a reciprocal relation is also evident between the conidial population and SD (Brown et al., 2008), prompting a balance between sporogenesis and SD in *Aspergillus* species. Hence, we associated the relatively lower abundance of fatty acids in 7 days incubated S1_VMI_ extracts with predominant mycelial forms. Conversely, the relative higher abundance of fatty acids in 7 days incubated S1 extracts might have triggered its early SD stage (Figure 3B). The oxylipins compounds, chemically the oxygenated fatty acids, are crucial metabolic cues governing inter- or intra-cellular signaling in plants, animals, as well as fungi (Tsitsigiannis and Keller, 2007). In our study, we observed a time correlated reciprocal relationship for the relative oxylipin levels *per se* between S1_VMI_ and S1 during the post-SD stages. Notably, a higher abundance of oxylipins was observed concomitant to the delayed SD stages (9 and 11 days) in S1_VMI_ (Figure 4B). In general, the secondary metabolite production commences in the late log-phase or resting stationary phase in microbes, including molds (Bu'Lock, 1961). Hence, although there is no conspicuous morphological distinction between the post-SD S1_VMI_ and S1 samples, there is a categorical distinction in the number and density of developed sclerotia. The plate morphology exhibits a relatively sparse distribution of pigmented sclerotia in S1_VMI_ compared to S1, following the 9 days incubation (Figure 2A). Typically, the oxylipins are characterized as the molecules for quorum sensing, promoting conidia at higher concentration and sclerotia at low concentration, within ambiguous critical limits (Brown et al., 2008). Further, we conjecture that the higher relative abundance of most oxylipin compounds detected during the late SD stages in S1_VMI_ might have accumulated due to the temporal delay in SD during the antecedent incubation stages.

In congruence to the oxylipin production patterns, the 13-desoxypaxilline abundance was also higher in S1_VMI_ only during the post-SD stages (Figure 4B). Previously, 13-desoxypaxilline production, an alfatrem precursor (tremorgenic mycotoxin), has been reported from *A. oryzae* RIB 40 cultivation on WATM agar (Rank et al., 2012). In *A. flavus*, the 13-desoxypaspalline and alfatrem biosynthesis are typically enhanced under the elevated oxidative stress, suggesting a stress responsive mechanism of molds (Fountain et al., 2016). Hence, by analogy the 13-desoxypaxilline production concomitant to the post-SD stages in S1_VMI_ might be associated with its oxidative stress response influencing its morphological transformation. Most of the non-identified (N.I) secondary metabolites follow a similar trend. Conversely, the relative abundance of an anthraquinone furan derivative i.e., UCT 1072M1 antibiotics, was relatively higher in S1 compared to S1_VMI_ during the SD (7 days) and post-SD (9 and 11 days) stages, respectively (Figure 4B). Recently, the coordination between fungal SD and secondary metabolism has been appraised and reviewed, highlighting the inextricable importance of *velvet* nuclear proteins (VeA and LaeA) in regulating the two apparently distinct phenomena (Calvo and Cary, 2015). Especially, *veA* genes are proposed vital for SD, but their function also depends on light exposure which impedes SD in sexually reproducing *Aspergillus* species. Hence, the higher abundance of UCT 1072M1 antibiotics might be justified through considering its twined metabolism with SD in *A. oryzae*. Though it will be difficult and perhaps imprudent to theoretically correlate the metabolic profiles with morphological phenotypes in fungi, these metabolomic trends can certainly pave the way for probing the associated biomolecular mechanism in a more generic way.

In recent years, fungal VOCs were suggested for influencing the plant growth modulation (Ditengou et al., 2015; Lee S. et al., 2016), induction of novel metabolites in prokaryotic symbionts (Schmidt et al., 2017), or antagonistic interactions with competing fungi sharing a common niche (Hiscox et al., 2015; El Ariebi et al., 2016) etc. Herein, we observed that intra-species VMI impeded mycelial growth rates in S1_VMI_ compared to control group S1, particularly during the SD and post-SD stages, with remarkably higher abundance of C-8 VOCs (Table 2). Previously, Yin et al. (2015) have reported that the common headspace VOCs *viz*., 1-octene-3-ol, 1-hexanol, and (E)-2-hexenal exhibits the growth inhibitory effects. Especially, the C-8 volatile compounds (a class of short chain volatile oxylipins), are known to inhibit growth and sporulation among receptive mold species through reversibly affecting a number of physicochemical parameters in fungal cell *viz*., membrane permeability, respiration, intracellular pH, and protein composition (Chitarra et al., 2005). Further, the authors have also reported the conspicuous effects of C-8 volatiles (1-octene-3-ol) on essential metabolic processes. Recently, we have also substantiated the similar growth inhibitory effects of headspace VOC's, including mostly the C-8 compounds, in *A. oryzae* strain (KCCM 60345) subjected to VMI with another ecotype *A. oryzae* strain (Singh and Lee, 2017). Although, there are no direct evidence correlating the biomolecular mechanisms vital for SD and VOCs emanated from an ecotype strain in filamentous fungi, we conjecture VOCs of particular class (C-8 compounds) somehow maneuver stress environ interfering this essential morpho-transformation in molds.

Recapitulating the available information about sclerotial biology in *Aspergillus* species, it is established that sclerotia are restive stages materialized by molds to escape or survive the environmental odds. Though the importance of SD in fungal dissemination, infection, and toxin productions are long been recognized, the vital metabolomic events as well as exogenous perturbations maneuvering this morphological phenomena are elusive. In the present study, we demonstrated an intertwined cascade of metabolic events unfolded during the categorical SD in *A. oryzae* RIB 40 subjected to VMI with partner strain in twin plate assembly. We illuminated a clear disparity in exometabolomes concomitant to SD, mycelial growth, media pH, and biochemical phenotypes in the S1_VMI_ strains as compared to its control strains (S1), underpinning the infochemical function of VOCs for intra-species interactions. We assert that both primary and secondary metabolism regulate as well as in-turn co-regulated in synergy during the course of morphological transformation in molds, affected by confounding factors including the VOCs.

## Author contributions

Design of research: DS and CL. Research work, analyses, and interpretation of data: DS. Manuscript writing: DS with conceptual directions from CL. Both authors approved the final version of the manuscript.

### Conflict of interest statement

The authors declare that the research was conducted in the absence of any commercial or financial relationships that could be construed as a potential conflict of interest.

## Abbreviations

SD: Sclerotia development
VOCs: Volatile organic compounds
VMI: VOCs mediated interactions
PCA: Principal component analysis
GC-TOF-MS: Gas chromatography time-of-flight mass spectrometry
UHPLC-LTQ-IT-MS/MS: Ultra high-performance liquid chromatography linear trap quadrupole ion trap tandem mass spectrometry
UPLC-Q-TOF: Ultraperformance liquid chromatography—quadrupole—time of flight—mass spectrometry
HS-SPME-GC-TOF-MS: Headspace—solid phase microextraction—Gas chromatography time-of-flight mass spectrometry
PCA: Principal component analysis
PLS-DA: Partial least squares—discriminant analysis
OPLS-DA: Orthogonal projection to latent structures—discriminant analysis.
