# Supplementary material for: Volatiles Mediated Interactions Between Aspergillus oryzae Strains Modulate Morphological Transition and Exometabolomes
Source: Front Microbiol. 2018 Apr 4;9:628. doi: 10.3389/fmicb.2018.00628 (PMC5893800; doi:10.3389/fmicb.2018.00628)
Supplement: Supplementary file 1 [file DataSheet1.DOCX]

***Supplementary Material***

**Volatiles Mediated Interactions between *Aspergillus oryzae* Strains Modulate Morphological Transition and Exometabolomes**

**Digar Singh, Choong Hwan Lee^*^**

Department of Bioscience and Biotechnology, Konkuk University, 05029, Seoul, Korea

*** Correspondence:**

Telephone: (+82) 220496177; Fax Number: (+82) 24454291; E-mail: chlee123@konkuk.ac.kr

**Supplementary Tables**

**Table S1.** Modified WATM (Wickerman's Antibiotic Test Medium) agar, originally adapted from Wickerman (1951).

| **S. No.** | **Components** | **Quantity (g/L)** |
| --- | --- | --- |
| 1 | Yeast extract | 2 |
| 2 | Peptone | 3 |
| 3 | Dextrose (D-Glucose) | 1 |
| 4 | Sucrose | 20 |
| 5 | Corn steep solid | 5 |
| 6 | Sodium nitrate | 2 |
| 7 | Potassium phosphate dibasic trihydrate | 1 |
| 8 | Magnesium sulfate | 0.5 |
| 9 | Potassium chloride | 0.2 |
| 10 | Ferrous sulfate heptahydrate | 0.01 |
| 11 | Agar (added after adjusting pH) | 20 |
|  |  |  |
| pH ~5.0 | | |

**Reference**

Wickerman, L. J. (1951). In *Taxonomy of yeasts, US Department of Agriculture Technical Bulletin No.*1029: 1–56. Washington, DC: US Department of Agriculture.

**Table S2.** The list of putatively identified significantly discriminant metabolites (VIP>0.7, *p*<0.05) based on PLS-DA datasets obtained using the temporal GC-TOF-MS analysis of *A. oryzae* RIB 40 (S1_VMI_ and S1) sample extracts.

| **S. No.** | **RT (min)** | **Metabolites** | **MS^n^ fragments** | **Unique ion (m/z)** | **TMS** | **Identity** |
| --- | --- | --- | --- | --- | --- | --- |
| ***Organic acids*** | | | | | | |
| 1 | 5.16 | Lactic acid | 73, 117, 147, 66, 75, 191, 190 | 117 | 2 | STD |
| 2 | 7.60 | Succinic acid | 147, 73, 149, 247, 129, 74, 172 | 247 | 2 | NIST |
| 3 | 7.88 | Fumaric acid | 73, 147, 241, 245, 113, 75, 255 | 245 | 2 | NIST |
| 4 | 9.18 | Malic acid | 73, 147, 75, 233, 133, 148, 101 | 233 | 3 | NIST |
| 5 | 10.69 | Kojic acid | 73, 147, 271, 132, 197, 231, 188 | 271 | 2 | STD |
| ***Amino acids*** | | | | | | |
| 6 | 5.54 | Alanine | 116, 73, 147, 75, 117, 59, 103 | 116 | 2 | STD |
| 7 | 6.68 | Valine | 144, 73, 145, 218, 147, 100, 75 | 144 | 2 | STD |
| 8 | 7.44 | Isoleucine | 158, 73, 142, 159, 147, 100 | 158 | 2 | STD |
| 9 | 7.50 | Proline | 73, 142, 73, 147, 75, 143, 148 | 142 | 2 | STD |
| 10 | 7.59 | Glycine | 147, 73, 148, 174, 55, 149, 247 | 174 | 3 | STD |
| 11 | 8.07 | Serine | 73, 188, 100, 204, 147, 59, 218 | 204 | 3 | STD |
| 12 | 8.31 | Threonine | 73, 117, 219, 218, 101, 147, 75 | 219 | 3 | STD |
| 13 | 9.38 | Pyroglutamic acid | 156, 73, 147, 157, 75, 59, 84 | 156 | 2 | NIST |
| 14 | 9.44 | Aspartic acid | 73, 232, 100, 147, 75, 156, 233 | 232 | 3 | STD |
| 15 | 9.52 | GABA | 73, 174, 156, 147, 86, 59, 175 | 174 | 3 | STD |
| 16 | 10.24 | Glutamic acid | 73, 246, 128, 147, 75, 156, 84 | 246 | 3 | STD |
| 17 | 10.34 | Phenylalanine | 73, 218, 192, 100, 147, 219, 75 | 218 | 2 | STD |
| ***Sugars and sugar alcohols*** | | | | | | |
| 18 | 7.24 | Glycerol | 73, 147, 103, 117, 205, 158, 133 | 205 | 3 | NIST |
| 19 | 10.64 | Xylose | 73, 103, 217, 147, 75, 74, 133 | 217 | 4 | STD |
| 20 | 11.06 | Xylitol | 73, 103, 217, 147, 129, 205, 117 | 217 | 5 | STD |
| 21 | 12.12 | Fructose | 73, 147, 217, 104, 205, 306, 364 | 306 | 5 | STD |
| 22 | 12.50 | Galactose | 73, 147, 103, 205, 319, 129, 75 | 319 | 5 | NIST |
| 23 | 12.58 | Glucitol | 73, 147, 205, 217, 319, 117, 74 | 319 | 6 | NIST |
| 24 | 12.86 | Mannopyranose | 73, 204, 191, 217, 75, 103, 205 | 204 | 5 | NIST |
| 25 | 13.61 | Myo-inositol | 73, 147, 217, 305, 191, 75, 103 | 305 | 6 | NIST |
| ***Fatty acids*** | | | | | | |
| 26 | 13.14 | Palmitic acid | 73, 117, 75, 132, 129, 145, 313 | 132 | 1 | STD |
| 27 | 13.44 | Oleanitrile | 55, 69, 122, 83, 436, 70, 57 | 122 | 1 | STD |
| 28 | 14.17 | Linoleic acid | 73, 75, 95, 117, 129, 262, 337 | 337 | 1 | STD |
| 29 | 14.33 | Stearic acid | 73, 117, 132, 129, 118, 145, 341 | 341 | 1 | STD |
| 30 | 15.33 | Oleamide | 75, 131, 73, 144, 116, 55, 128, 69 | 131 | 1 | STD |
| ***Others*** | | | | | | |
| 31 | 5.69 | Hydroxylamine | 73, 133, 146, 119, 59, 147 | 133 | 3 | NIST |
| 32 | 6.87 | Pentanoic acid | 73, 147, 103, 159, 75, 79, 171 | 159 | 2 | NIST |
| 33 | 7.19 | Phosphoric acid | 73, 158, 299, 147, 74, 59, 300 | 299 | 3 | NIST |
| ***Non-identified (N.I)*** | | | | | | |
| 34 | 5.26 | N.I. 1 | 79, 75, 147, 52, 117, 77, 59 | 147 | NA | NA |
| 35 | 8.46 | N.I. 2 | 73, 373, 147, 57, 100, 258, 71 | 100 | NA | NA |
| 36 | 10.00 | N.I. 3 | 73,147, 193, 220, 91, 194, 75 | 193 | NA | NA |
| 37 | 10.39 | N.I. 4 | 73, 179, 164, 75, 252, 149, 296 | 252 | NA | NA |

**RT:** Retention time; **TMS:** trimethylsilyl units; **ID:** identification; **STD:** standard compounds, **NIST:** (national institute of standards and technology library), **NA** - not applicable. **S1_VMI_:** Strain 1 (*A. oryzae* RIB 40, KACC 44967) subjected to VOC's mediated interaction with partner strain, S2 (*A. oryzae*, KCCM 60345); **S1:** Strain 1 (*A. oryzae* RIB 40, KACC 44967) cultivated without partner strain S2, in twin plate assembly.

**Supplementary Figures**

**
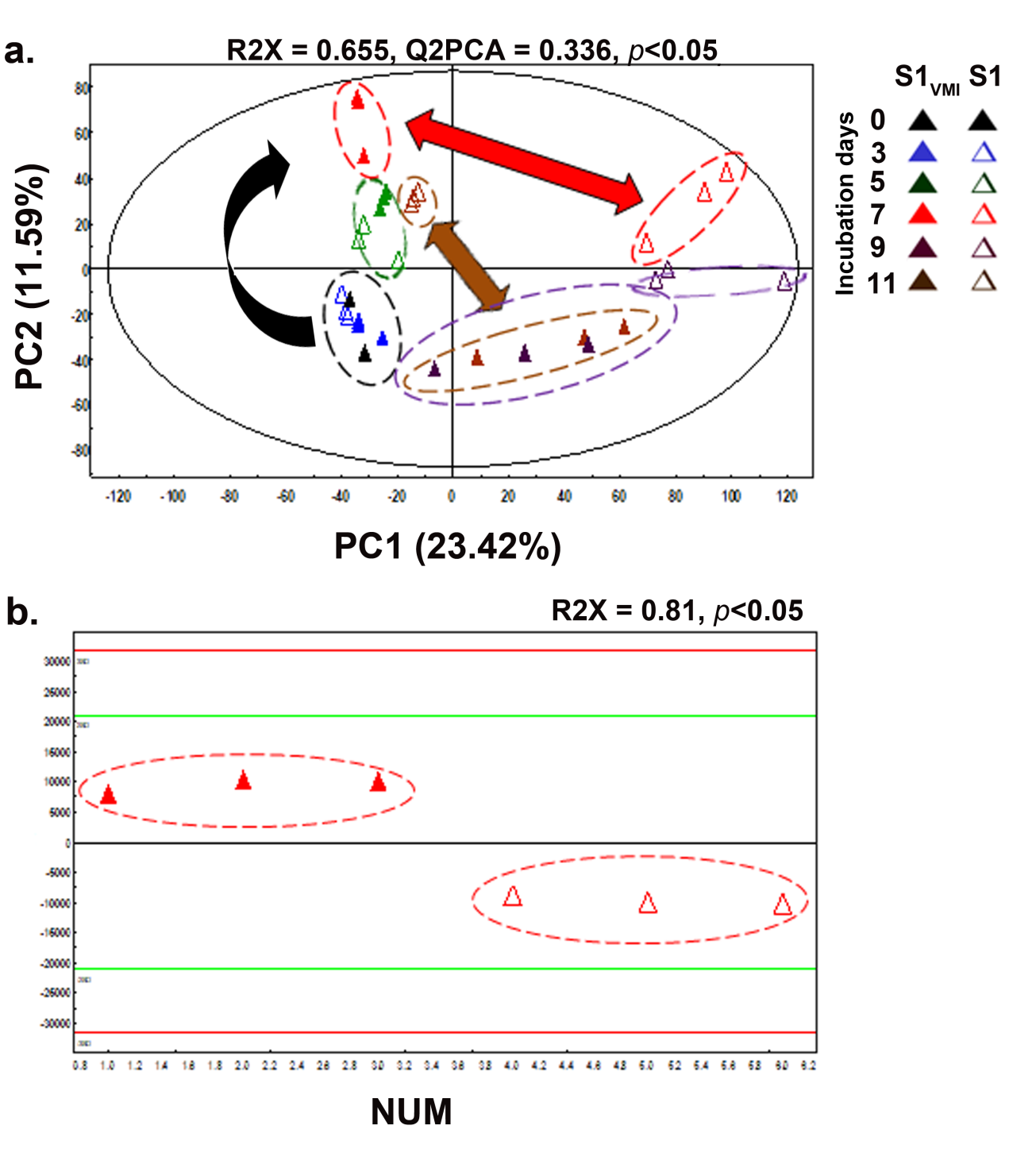
**

**Figure S1. (a)** The PCA score plot displaying the time correlated (0 to 11 days) trajectory of metabolomic variability, and **(b)** the OPLS-DA score plot indicating the metabolic disparity between the 7 day incubated, *A. oryzae* RIB 40 (S1_VMI_ and S1) sample extracts based on GC-TOF-MS derived datasets. Herein, the colored symbols signifying the respective incubation days are indicated in the figure inset. **S1_VMI_:** Strain 1 (*A. oryzae* RIB 40, KACC 44967) subjected to VOC's mediated interaction with partner strain, S2 (*A. oryzae*, KCCM 60345); **S1:** Strain 1 (*A. oryzae* RIB 40, KACC 44967) cultivated without partner strain S2, in twin plate assembly**.**

**
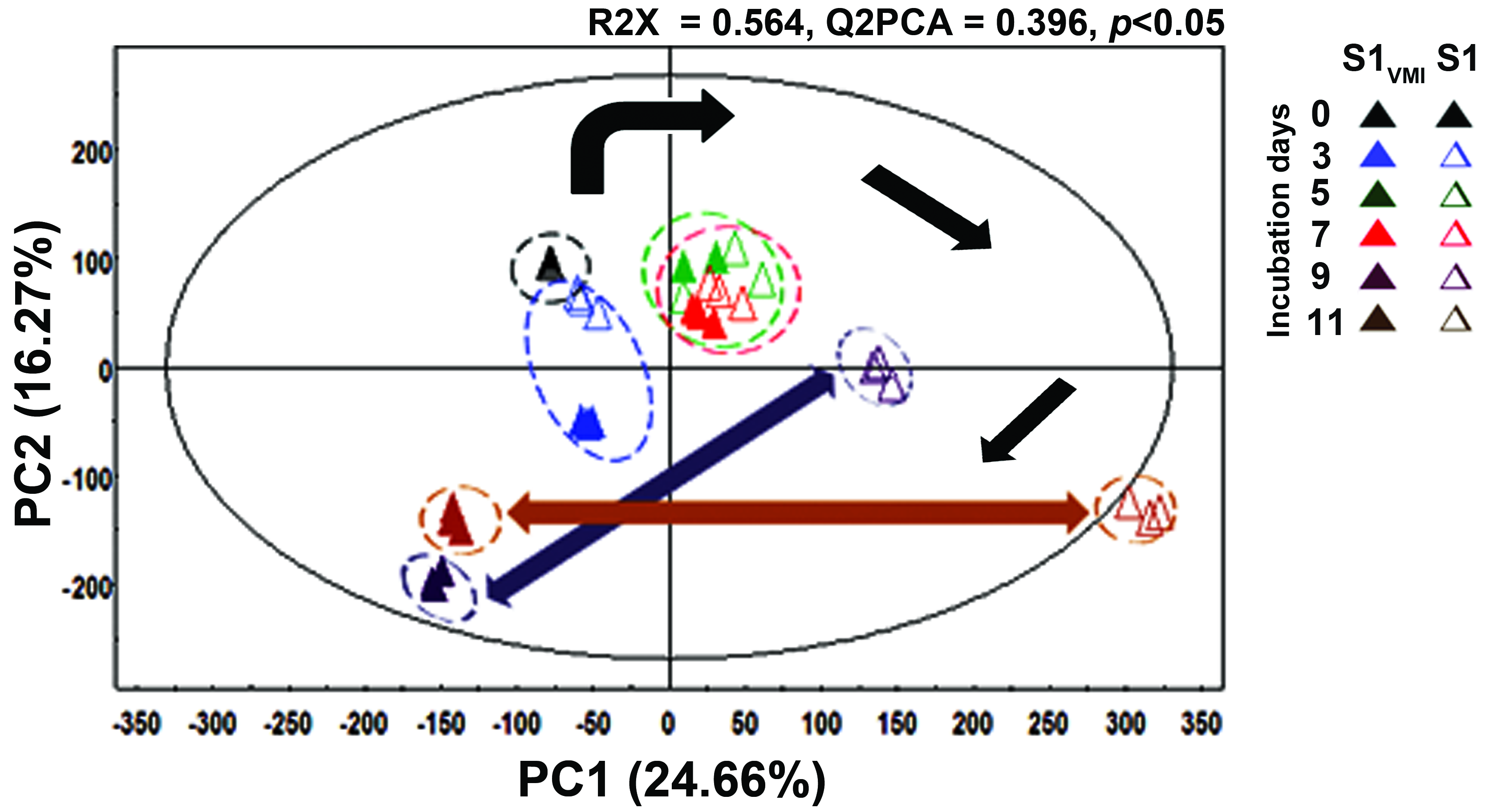
**

**Figure S2.** The PCA score plot displaying the time correlated trajectory of metabolomic variability for *A. oryzae* RIB 40 (S1_VMI_ and S1) sample extracts based on UHPLC-LTQ-IT-MS/MS analysis datasets. Herein, the colored symbols signifying the respective incubation days are indicated in the figure inset. **S1_VMI_:** Strain 1 (*A. oryzae* RIB 40, KACC 44967) subjected to VOC's mediated interaction with partner strain, S2 (*A. oryzae*, KCCM 60345); **S1:** Strain 1 (*A. oryzae* RIB 40, KACC 44967) cultivated without partner strain S2, in twin plate assembly**.**
